# A randomised controlled trial examining the efficacy of smoking-related response inhibition training in smokers: a study protocol

**DOI:** 10.1186/s12889-018-6109-y

**Published:** 2018-11-03

**Authors:** Petra K. Staiger, Melissa J. Hayden, Karen Guo, Laura K. Hughes, Jason Bos, Natalia S. Lawrence

**Affiliations:** 1 School of Psychology, Deakin University, Geelong, VIC 3220 Australia; 2 Centre for Drug Use, Addictive and Anti-social behaviour Research (CEDAAR), Deakin University, Locked Bag 20000, Geelong, VIC 3220 Australia; 3 Cognitive Neuroscience Unit, School of Psychology, Deakin University, 221 Burwood Hwy, Burwood, VIC Australia; 4 Department of Psychology, University of Exeter, Perry Road, Prince of Wales Road, Exeter, EX4 4QG UK

**Keywords:** Smoking cessation, Response inhibition, Inhibitory control, Cognitive training, Devaluation, eHealth, Craving, Intervention

## Abstract

**Background:**

Smoking is one of the leading preventable causes of illness and premature death worldwide. Despite a variety of effective treatments, relapse rates remain high, and novel, innovative interventions are needed in order to reduce the global prevalence of smoking. Research has indicated that deficits in the ability to inhibit a response (referred to as response inhibition) is a predictor of relapse and subsequently, targeting this potentially modifiable risk factor may lead to improvements in smoking outcomes. Indeed, in recent years, stimulus-specific response inhibition training has emerged as a potentially efficacious intervention to reduce unwanted/unhealthy behaviours such as alcohol and unhealthy food consumption. As such, the present trial is the first to evaluate the real-world efficacy of response inhibition smoking training (INST) in a sample of adult heavy smokers.

**Methods/design:**

This randomised controlled trial will recruit nicotine dependent smokers aged between 18 and 60 using social media and advertisements in Victoria, Australia. The sample target was 150 to account for drop out and non-adherence. Once informed consent has been obtained, participants complete a range of baseline measures during a face to face interview. Participants are randomly allocated to one of two online training conditions: an intervention training group (INST), which requires participants to exercise response inhibition towards smoking-related stimuli; or an active control group, which requires participants to exercise response inhibition towards household items and does not include any smoking-related stimuli. They complete the first training session during the interview to ensure the training protocol is clear. Both groups are instructed to complete a further 13 training sessions (1 per day) at home on their computer and follow-up phone calls will be conducted at three time points: post-intervention, one-month and three months. The primary outcomes are: a) rates of smoking cessation and; b) reduction in the quantity of average daily smoking at post-intervention, one and three months follow-up.

**Discussion:**

There is a pressing need to develop novel and innovative smoking interventions. If proven to be effective, INST could make a highly cost-effective contribution to improvements in smoking intervention outcomes.

**Trial registration:**

The trial was prospectively registered with the Australian New Zealand Clinical Trials Registry 17th February 2017. Trial ID: ACTRN12617000252314.

**Electronic supplementary material:**

The online version of this article (10.1186/s12889-018-6109-y) contains supplementary material, which is available to authorized users.

## Background

Tobacco smoking is one of the leading preventable causes of illness and premature death worldwide. It is the second largest contributor to the burden of disease globally, with 134.2 million years lost to disability, illness and premature death [1]. In 2013, 6.1 million people died globally as a result of tobacco use [1], and, if trends persist, this number will exceed 8 million by the year 2030 [2]. Furthermore, tobacco use has been found to cost the global economy more than US$1 trillion each year in healthcare expenditures and lost productivity [3]. As smoking remains at unacceptable levels across the world [4, 5], examinations of effective and accessible smoking cessation treatments are crucial in reducing the global burden of smoking on public health.

Currently, pharmacological and psychosocial interventions have the most support as efficacious treatments for the cessation of smoking [6–10]. However, despite the positive outcomes associated with these interventions, most smokers do not seek formal treatment to reduce smoking [11] and existing treatments can entail several limitations. First, smokers have reported concerns regarding adverse side-effects of pharmacological treatments which have impacted treatment uptake and long-term adherence [12–14]. Second, the long-term cost of pharmacological and psychosocial interventions, which can be more expensive than cigarettes themselves, often prohibit individuals from accessing smoking cessation treatments. This is particularly relevant given that: 1) the financial costs of tobacco are one of the primary reasons underpinning quit intentions and attempts [4, 15, 16] and; 2) the incidence of smoking is increasing most rapidly in developing nations who have the lowest levels of disposable income [5]. Therefore, there is a critical need for accessible and cost-efficient interventions for smoking cessation. Third, relapse rates remain consistently high following treatment [17] and, the vast majority will relapse within five to 10 days of treatment cessation [18, 19]. Thus, a substantial proportion of individuals attempting to quit smoking fail to achieve long-term abstinence, inviting the question: what modifiable risk factors for smoking relapse may be targeted to increase abstinence rates or at the very least result in reduction of level of smoking?

Previous research indicates that deficits in response inhibition are a strong predictor of relapse for smokers following a quit attempt [20, 21]. Research suggests that recently abstinent smokers experience heightened difficulties with response inhibition [22, 23], indicating that targeting this may assist in preventing relapse. Importantly, a meta-analysis [23] supports evidence showing that individuals dependent on substances such as cocaine and alcohol may experience deficits in response inhibition. Furthermore, Yin and colleagues [24] found that a group of smokers reported response inhibition deficits on the GNG task. Taken together this provides some evidence that smokers may experience difficulties with response inhibition. Of significance is that individuals who reported higher nicotine dependence experienced greater deficits in response inhibition than those of lower use or dependence [25, 26]. Given that heavier smokers find it more difficult to quit [27, 28], response inhibition deficits may be an effective target for treatment in these individuals.

Indeed, response inhibition training interventions utilising tasks such as the go/no-go (GNG) task and stop signal task (SST) focus on training successful inhibition of a habitual or pre-potent response by pairing pictorial cues of the targeted behaviour with stop signals or no/go cues [29]. The GNG task targets automatic bottom-up response inhibition (or action restraint) by consistently pairing no-go cues with the target stimuli [30, 31], while the SST targets top-down inhibitory control (or action cancellation) as stop signals occur after an initiated response and are mapped only to a proportion of target stimuli [32]. These tasks have recently been examined to reduce alcohol and food intake, yielding efficacious results [33]. For example, Houben, Havermans, Nederkoorn, and Jansen [34] randomly assigned 57 heavy alcohol drinkers to receive one of two training conditions: a beer/no-go condition, where alcohol-related stimuli were consistently paired with a stopping response, or a beer/go condition, where participants always responded to alcohol-related stimuli. Compared to participants in the beer/go condition, those who were trained to inhibit their response towards alcohol-related stimuli (beer/no-go) reported significantly less alcohol intake. Similar findings were reported by Jones and Field [35]. In their study, following motor inhibition training utilising a modified SST, heavy social drinkers were found to consume significantly less alcohol in a subsequent ad libitum taste test.

More recently Lawrence et al. [36] implemented an internet-delivered response inhibition training intervention for food among 83 overweight and obese adult participants. Participants were randomly allocated to receive four 10-min training sessions completed online. In the intervention group, high-calorie foods were consistently paired with no-go signals and in the control group, non-food stimuli were consistently paired with no-go signals. At one-week follow-up, participants in the food no-go condition consumed significantly less food, showed significant weight loss, and had decreased positive evaluations towards high calorie foods compared to controls. At 6 month follow-up, participants in the intervention group displayed significantly higher average weight loss (2.21 kg) compared to controls (0.36 kg). These findings are consistent with a previous trial [37] that compared two interventions for losing weight: an implementation intention intervention that instructed participants to plan reminders for dieting and a response inhibition intervention that paired no-go responses with food-related stimuli. Findings indicated that participants who completed only the response inhibition training reported significant weight loss after four training sessions. Together, these results indicate that response inhibition training can be effectively delivered online, promoting greater accessibility and cost-efficiency of these types of interventions.

Two meta-analyses have found that inhibitory control training resulted in an overall significant effect (albeit a small effect size), with GNG training yielding larger (medium) effect sizes than SST training [29, 33]. According to the Behaviour Stimulus Interaction (BSI) theory [38] behavioural changes induced by the GNG training are mediated by changes in evaluations of the stimuli used in the task. That is, positively regarded stimuli will become associated with negative affect as a result of consistently being paired with no-go cues. This is thought to devalue the stimuli and minimise the likelihood of approach behaviours occurring towards the stimuli in real life. This theory has been supported by evidence in studies targeting alcohol consumption that suggest a mediating effect of changes in implicit attitudes on alcohol intake [34, 39]. In the food domain, there is evidence of devaluation of trained no-go food stimuli as assessed by visual analogue scales [36, 40, 41]. Another proposed mechanism of response inhibition training is the automatic inhibition hypothesis (AIH) [32], which posits that automatic response inhibition can develop over practice if stimuli are consistently associated with stopping [42, 43]. These two potential mediating hypotheses will be investigated in this trial.

In summary, given that significant effects of the GNG task were found despite the use of non-clinical samples, it was expected that these interventions would be particularly effective with smokers as smoking receives the most frequent reinforcement compared with other dependent populations, with multiple smoking sessions each day. Furthermore, we hypothesise that it will be particularly beneficial for heavy smokers who report the greatest difficulty with impulse control [4]. This is suggested by findings that stronger nicotine dependence is associated with poorer inhibitory control [44]. Thus, this is the first study to use the GNG task in a sample of individuals who have a Tobacco Use Disorder according to DSM-5 criteria and who wish to quit/reduce smoking.

As previous studies have found response inhibition training to be effective even when administered over the internet [36, 37], this study delivered the training paradigm online. This enabled the intervention to be accessible, convenient and cost-efficient for individuals and further contribute to reducing the burden on other treatment services and resources. The study is a randomised controlled trial (RCT) examining the efficacy of response inhibition training in reducing smoking in heavy dependent smokers. It is implemented in accordance with CONSORT guidelines, and involves collecting follow-up data from participants at 1 month and 3 months post-intervention.

### Primary hypotheses

1. Smokers who received smoking-related response inhibition training (INST program) would report significantly higher cessation rates compared to those in the active control condition at the end of the intervention, 1 month and 3 months post-intervention.
2. Smokers who received smoking-related response inhibition training (INST program) would report significantly less cigarette consumption compared to smokers in the active control condition at the end of the intervention, 1 month and 3 months post-intervention.

### Secondary hypotheses

1. Smokers who received smoking-related response inhibition training (INST program) would report significantly less craving for cigarettes compared to smokers in the active control condition at the end of the intervention and 1 month and 3 months post-intervention.
2. Smokers who received smoking-related response inhibition training (INST program) would report significantly lower levels of nicotine dependence compared to smokers in the active control condition at the end of the intervention and 1 month and 3 months post-intervention.

### Predictor/moderator hypotheses

1. Individuals reporting high levels of impulsivity would report significantly improved outcomes from the intervention training compared to those with lower levels of impulsivity^.^
2. Individuals who completed a greater number of sessions (i.e., dose) would report significantly improved outcomes from the intervention training compared to those who completed less sessions.

### Mediator hypotheses

1. The effects of INST training on level of smoking would be mediated by devaluation of smoking stimuli as measured by a devaluation of smoking images task.
2. The effects of INST training on level of smoking would be mediated by an independent measure of response inhibition (SST).

The following exploratory question was proposed:

1. Do smokers who receive smoking-related response inhibition training (INST program) report significantly higher levels of self-confidence and motivation to quit smoking compared to smokers in the active control condition at the end of the intervention, 1 month and 3 months post-intervention.

## Methods/design

### Design

This is a 2-group parallel-block double-blind randomised controlled trial testing the efficacy of an intervention compared to an active control training. The intervention training is a smoking version of the food GNG training task in Lawrence et al. [36]. The active control training is similar to the control training in Lawrence et al. [36], with no-go training to household items. The Deakin University Human Research Ethics Committee (DUHREC) reviewed and approved all relevant study materials (Project ID: 2015–298). The trial was registered with the Australian New Zealand Clinical Trials Registry (Trial ID: ACTRN12617000252314; see Additional file 1: Table S1 for items from the World Health Organisation Data Set as per Spirit Guidelines). No study protocol amendments were made once the trial commenced and this protocol was originally submitted to this journal 1 November 2017.

### Procedure

The following sections describe the study procedure. See Table 1 for an overview.

#### Initial screening

Participants were adult smokers aged between 18 and 60 years, recruited through social media and advertisements in Victoria, Australia who had a desire to quit smoking.

#### Inclusion criteria

- Aged between 18 and 60 years.
- Smoke, on average, a minimum of 10 cigarettes per day for the last 12 months.
- Meet criteria for moderate or above Tobacco Use Disorder defined by the DSM-5 [45].
- Be motivated to make a quit attempt during the training stage of the intervention.
- Completed at least Year 9 (or equivalent) schooling.
- Have computer and internet access during the intervention phase of the study.

#### Exclusion criteria

- Primarily uses electronic cigarettes on a daily basis.
- Non-smoking period of 2 weeks or more in the past 3 months.
- Currently using anti-craving medication.
- Using nicotine-replacement therapy during the intervention period.
- Self-reported problematic alcohol or drug(s) use other than tobacco.
- Reported a traumatic or acquired brain injury or a loss of consciousness for more than 30 min.
- Reported current use of psychotropic medication such as anti-depressant, anti-psychotic and/or anxiolytic medication.

Interested participants were invited to contact the research team via email. They were screened over the phone/online to determine their eligibility. Participants who met the inclusion criteria were invited to participate in the study and attended a face to face interview in order to sign the consent form, collect baseline measures and participate in the first online training session.

#### Baseline assessment (T1)

At the beginning of the baseline interview session, participants read the plain language statement and if in agreement signed the consent form. They were requested to report any adverse events or consequences which will be reported in the flow chart of the primary outcomes paper. They were informed that they were able to withdraw from the study at any time. They were asked to indicate whether they would like to receive a summary of the trial findings following completion of data analyses. Participants were informed that they would receive one of two brain training tasks as the aim of the study was to investigate which one was more effective. While they were informed that the task incorporated a “variety of visual images”, the types of images were not specified to prevent participants from identifying if they were in the control group and hence we propose that participants were likely blind to the nature of the intervention and whether they were randomised to an active condition.

Participants completed a battery of questionnaires (outlined in Table 1), and completed ratings of their craving, motivation and self-efficacy. Following the completion of the questionnaires, participants completed ratings of stimulus evaluation test and a smoking stop signal task (SST), an independent measure of response inhibition separate to the response inhibition training.

**Table 1:** SPIRIT Flow Diagram of the schedule for participants and data collection for the INST study

		STUDY PERIOD					
				Follow-Up Period			
	Enrolment	Baseline (T1) and Allocation	Training Period	Post-Intervention (T2)	1-Month Post-Intervention (T3)	3-Months Post-Intervention (T4)	Close-out
TIMEPOINT	*-t*_*1*_	*t*_*0*_ *-t*_*1*_	*t*_*2*_ *– t*_*14*_	*t*_*15*_	*t*_*45*_	*t*_*105*_	*t*_*x*_
ENROLMENT:							
Eligibility screen	X						
Informed consent	X	X					
*Obtain Contact Information*	X	X					
Allocation		X					
INTERVENTIONS:							
*INST intervention*		X	X				
*Control training*		X	X				
ASSESSMENTS:							
*Demographic Questions*		X					
*TLFB*		X		X	X	X	
*FTND*		X		X	X	X	
*DASS*		X		X	X	X	
*AUDIT*		X				X	
*BIS-11*		X		X	X	X	
*Stimulus Evaluation Test*		X		X	X	X	
*SST*		X		X		X	
*Craving Rating*		X	X	X	X	X	
*Motivation Rating*		X		X	X	X	
*Confidence Rating*		X		X	X	X	
*Time of last cigarette*		X	X				
CLOSE-OUT:							X
*Data-analysis*							X
*Debriefing of participants*							X
*Documentation and Dissemination of Findings*							X

t refers to days (from t_1_ onwards). *TLFB* Timeline Follow-Back interview, *FTND* Fagerström Test of Nicotine Dependence, *DASS* Depression Anxiety and Stress Scale, *AUDIT* Alcohol Use Disorders Identification Test, *BIS-11* Barrett Impulsiveness Scale, *SST* Stop Signal Task

#### Randomisation

Immediately following the completion of the baseline assessment, participants began the online training task. Participants were automatically randomised to either the intervention or the control training task via a pre-computed randomisation procedure. A permuted block randomisation procedure was utilised [46] whereby participants were allocated to the intervention or control group through the use of a randomly generated number. The permuted blocks were organised in groups of ten, the details of which were not known by investigators involved with the administration of the trial. The use of the permuted block randomisation process ensures that intervention group numbers will be balanced at the end of each block and is thus the recommended process in studies with smaller samples.

Upon finishing this task participants were instructed to complete the online training task once per day for the next 13 days, totalling 14 sessions. They were asked to rate their smoking craving level before and after each training session. Twice per week, participants were sent text reminders to complete the training. All data from the online training task and outcome measures were securely stored on the Deakin University server and linked to an anonymous participant ID number such that only de-identified data were available to researchers. The data was checked for training task performance accuracy and participant adherence to the training protocol by a research assistant who was independent from investigators and not involved in data collection or analyses.

#### Inhibition training task

**Fig. 1:**
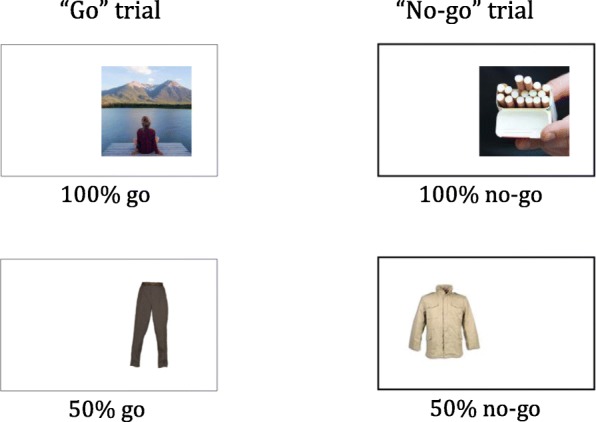
Overview of the “go” and “no-go” trials in the treatment condition of the GNG task. Image source (clockwise from top left) - permission granted: Pixabay [71]; Studio Art/Shutterstock [72]; Natalia S. Lawrence (Author)

The intervention is an online GNG training task as developed by Lawrence et al. [36], modified to incorporate images of smoking. The task included nine smoking images (or household items in the control group), nine relaxation images (or household items in the control group) and 18 neutral filler images presented on the left or right of the computer screen (see Fig. 1). Each image was presented for 1250 ms followed by a 1250 ms inter-stimulus interval. Participants were instructed to indicate whether the image is located to the left or the right of the screen using the keys “C” and “M” respectively on their keyboard. On half of the trials, the frame around the picture was bolded and the participants were required to not respond (no-go trials). On the other half of the trials the frame was not bolded (go trials) and the participant were required to respond as quickly as possible. During each training session participants completed 6 training blocks, with each of the 36 images presented once per block. At the end of each block, participants were provided with feedback on their accuracy and mean correct go reaction time and will be encouraged to continue trying to beat their own score. Each training session will last for approximately 10 min. Participants were asked to complete the training at home in a quiet place and preferably, when they experienced cravings for a cigarette.

#### Intervention group

The intervention consisted of nine smoking-related images, nine relaxing images.

(i.e. depicting relaxing/enjoyable activities), and 18 neutral filler pictures (e.g. clothing). For the intervention group, the smoking-related pictures were always “no-go” trials and the non-smoking pictures were always “go” trials. The neutral pictures were equally “go” and “no-go” trials (see Fig. 1). The neutral filler pictures were incorporated to prevent participants from easily identifying the associative rules of the task and to ensure the task remains challenging and engaging.

#### Control group

In the control group, participants complete a similar task to the smoking intervention group except that randomly presented 18 images of household objects replace the 18 smoking and relaxation images. The household images were presented equally as “go” and “nogo” trials.

#### Post-intervention (T2)

At the completion of the two-week intervention period, participants are contacted via phone by a researcher naïve to the group randomisation (i.e. a different researcher to the one who conducted the baseline interview). They receive a text message reminder 24-h prior to confirm the time of the phone call. During these phone interviews, participants are asked to provide details about their use of cigarettes and nicotine replacement therapies or anti-craving medications over the previous 2 weeks. At the conclusion of this interview, participants are emailed a link to complete the same battery of questionnaires, ratings of their craving, motivation and self-efficacy and SST (completed last) as completed at baseline (T1).

#### One-month and three-months follow-up (T3 and T4)

Follow-up at 1 month (T3) and 3 months (T4) are conducted in the same manner as T2. The two follow-up time points are identical with the exception that the SST was not completed at T3 only in T4 to reduce participant burden. At the completion of each time point, participants were mailed a $20 gift card. At the conclusion of the data collection period, participants in the control group are offered the opportunity to complete the smoking-related response inhibition training.

## Measures

This study used information from a face-to-face interview session (T1) and phone interviews (T2, T3 and T4), in addition to self-report questionnaires, a cognitive task and a stimulus evaluation test. A list of measures used at each assessment point is provided in Table 1. Demographic information, such as age, gender, socioeconomic status and number of years of smoking, were collected at baseline.

### Researcher-administered measures

#### *Timeline Follow-Back (TLFB)* [47, 48]

The TLFB is a calendar-based assessment of daily cigarette use for periods of time ranging from 1 to 12 months prior to assessment. Initially developed to assess alcohol consumption, the TLFB has since been utilised to assess a variety of substance use inclusive of cigarette use [47]. Memory aids are used to enhance recall of certain time-periods in order to retrospectively estimate number of cigarettes used for each date. The cigarette TLFB has shown high test-retest reliability and temporal stability across both clinical and non-clinical participants [47].

### Self-report measures

#### *Fagerström Test for Nicotine Dependence (FTND)* [49]

The FTND is a six-item self-report questionnaire of nicotine dependence. Dichotomous items (yes or no) are scored as 0–1, and options for categorical items are scored 0–3. The FTND has a maximum score of 10, with higher scores indicating greater nicotine dependence. The FTND demonstrates moderate internal consistency (α = .61) and has been validated in smokers from the general population [48] and in a clinical sample [50].

#### Craving for cigarettes

A one-item question utilising a 100 mm slider scale measures craving from “not at all” to “extremely”. Participants respond to the question “How much are you currently craving a cigarette”. A slider bar is presented at the left end of the scale and participants will click and drag the bar along the scale to indicate their response. It has been found that a single measure of craving is just as reliable and sensitive as self-report questionnaires for measuring craving for smoking [51, 52]. Slider scales are considered to be an engaging type of interface [53] and are regarded as a psychometrically acceptable measurement [54].

#### *Depression, Anxiety and Stress Scale (DASS-21)* [55]

The DASS-21 is a 21-item measure consisting of three subscales: depression, anxiety, and stress. Participants are asked to use a four-point Likert scale to rate the extent to which they have experienced the state described over the past week. The DASS has excellent internal consistency for the total scale (α = .97), and each subscale (Depression = .96; Anxiety = .92; Stress = .95) has high test-retest reliability and acceptable construct and convergent validity [56].

#### *Alcohol Use Disorders Identification Test (AUDIT)* [57]

The AUDIT is a 10-item measure of alcohol problems. Questions relate to frequency and quantity of consumption, and alcohol-related problems. Participants are asked to rate items from 0 to 4 and can receive a maximum possible score of 40, with higher scores indicative of more hazardous drinking, AUDIT is highly reliable and valid for use across a range of populations [58].

#### *Barratt Impulsiveness Scale (BIS-11)* [59]

The BIS-11 is a 30-item questionnaire assessing trait impulsivity. Each item is scored on a four-point Likert scale that ranges from “rarely/never” to “almost always”. Scores are summed to yield an overall total score ranging from 30 to 120, with higher scores indicating greater trait impulsivity. The BIS-11 also provides scores on three subscales: attentional impulsiveness, motor impulsiveness, and non-planning impulsiveness. The BIS-11 is widely used in research and clinical contexts and has been shown to demonstrate good reliability [59, 60].

#### Ratings of motivation and self-efficacy

Participants are asked to rate their motivation (“currently, how motivated are you to reduce or quit smoking?”) and self-efficacy (“currently, how confident are you in your ability to quit or reduce smoking?”) on slider scales. The scale is a 100 mm line with the left anchor labelled “not at all” and the right anchor labelled “extremely”. Similar to the craving slider scale, participants indicate their response by clicking and dragging the slider bar along the scale.

#### Stimulus evaluation test (ratings of likeability of smoking and relaxing images)

Slider scales are used for the likeability ratings of the smoking and relaxing images used in the inhibition training task (INST). Participants are presented with the question, “how much would you like to do this activity right now?” and rate the images from “not at all” to “extremely”. The slider bar is presented in the middle of the scale and participants click and drag the slider bar to indicate their response.

### Cognitive task

#### Stop Signal Task (SST) [32, 61, 62]

A smoking-specific version of the SST [30, 32, 61–63] is utilised. The SST contains images of smoking-related stimuli that are different images from those used in the intervention task. Participants are presented with a fixation cross in the centre of a screen on a white background for 500 ms. A smoking-related image (go-stimulus) then appears for 1000 ms, followed by a blank white screen for 1000 ms (inter-stimulus interval). The 16 images used in the SST are comprised of 8 pairs of images, where one image of the pair is a cigarette pointing to the left, and the second image is its mirror image pointing to the right. As such, the presentation of stimuli pointing left or right will be equally balanced. Each of the 16 images is presented a total of 12 times.

**Fig. 2:**
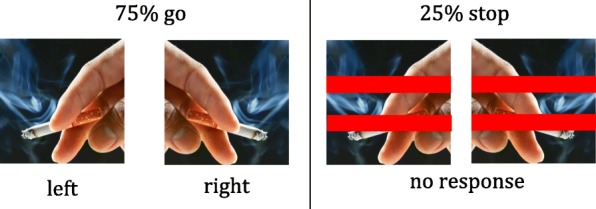
Overview of the “go” and “stop” trials in the Stop Signal Task illustrating correct responding. Image source - permission granted: Khamiranon D/Shutterstock [73]

Participants are instructed to indicate whether the cigarette is pointing left or right by pressing the computer keys “C” or “M” respectively (Fig. 2). The stop signal is a pair of red lines across the image and will appear on 25% of trials. It appears at a short delay (Stop Signal Delay or SSD) after the onset of the go stimulus and stays on screen until the inter-stimulus interval. Participants are instructed to respond as quickly as possible but to not respond when the red lines appear. This delay between the onset of the go signal and the stop signal begins at 250 ms on the first stop trial, and then adjusted by 50 ms in a staircase manner. Successful inhibition on stop trials results in the SSD increasing for the next stop trial, while unsuccessful inhibition, where the participant responds on a stop trial, will shorten this delay by 50 ms. The SST consists of one practice block of 10 trials followed by the experimental block of 192 trials. The SSD will be used to calculate the stop signal reaction time (SSRT) as a measure of response inhibition and the reaction time on go trials will be a measure of behavioural impulsivity [62].

### Analysis plan

All participants will be included in the intent-to-treat analyses for the primary and secondary hypotheses. If relevant, per protocol analysis will include those who complete at least four sessions of the training (as per Lawrence et al. [36]) and achieve a training accuracy of at least 70%. SST data will be included for those who yield an accuracy of 40–60% on stop trials and at least 70% on go trials. Prior to analyses, all variables will be examined through IBM Statistical Package for Social Sciences (SPSS Version 25) for accuracy of data entry, missing values and fit between their distributions and the assumptions of multivariate analysis. Any violations will be addressed as per standard protocols [64].

Missing data will be managed using SPSS. First, a missing value analysis will be conducted to determine the percentage and pattern of missing data. If missing data are found to relate to a measured participant variable, those variables will be included as covariates in the analyses. If appropriate, multiple imputation will be used to replace missing values and the imputation model will include baseline covariates and outcome data. Missing data will be imputed using the Markov Chain Monte Carlo method or the Monotone method, contingent upon the pattern of missing data. A minimum of five imputed datasets will be produced [65]; however, depending on the percentage of missing data, a minimum of 20 imputed datasets may be required [66]. Wherever possible, results from the complete case analysis will be compared with results based on imputed data. If there are important differences, explanations will be offered.

The primary and secondary hypotheses will be analysed using separate mixed-design ANOVAs and a Chi-square analysis for the binary outcome. Depending on the rate of smoking abstinence at follow up the outcome variable will be calculated as either binary (smoking abstinence: yes/no) or percent days abstinence if Chi-square analysis is contraindicated due to low numbers in each cell. For all other mixed design ANOVAs group (i.e., intervention or control) will be included as the between-subjects factor and time (survey time points) is the within-subjects factor. For the smoking reduction primary hypothesis, the repeated-measures factor will be the average number of cigarettes smoked per day at each timepoint (i.e., baseline, post-intervention, 1 month and 3 months post intervention). For the secondary hypotheses, the repeated-measures factor will be craving or nicotine dependence at each timepoint. The predictor hypotheses will be examined using separate moderated regression analyses, with group as the predictor variable, impulsivity and dose as the moderator variable and change in smoking as the dependent variable.

The two mediation hypotheses will be analysed utilising a linear mixed model approach to examine whether the effects of INST training on level of smoking will be mediated by devaluation of smoking stimuli or an independent measure of response inhibition (SSRT).

The exploratory questions related to self-confidence and motivation will be examined using a separate mixed-design ANOVA, with group included as the between subjects factor and self-confidence and motivation at each timepoint included as the repeated-measures factor.

Repeated measures ANOVAs will be performed on “go” reaction times and “no-go” accuracy to examine stimulus-specific learning effects (100% stimuli vs. 50% stimuli) over time (first vs. fourth training session as per the analysis by Lawrence and colleagues to allow comparability). Evidence of learning across the two time points will be indicated by faster reaction time on 100% go stimuli and fewer errors on 100% no-go stimuli. Any further exploratory analyses will be labelled as such in the publication.

#### Power analysis

As previous ICT studies have not targeted abstinence the current study was powered on smoking reduction based on Lawrence et al., [36] weight reduction ICT outcome data. Power analysis conducted via G*power indicated that an overall sample size of 92 is required to detect a medium effect size (approximately .50 cohen’s d based on Lawrence et al.) at the .05 alpha level using linear techniques (power = .80). Given that it is expected that approximately 25% will be lost to follow-up and up to 30% would not complete a minimum of 4 sessions, the target of the current study was set at 150 at the time of trial registration. However, estimated target sample may be amended if attrition is better than expected.

## Discussion

Despite a decline in smoking rates prevalence of tobacco smoking still remains unacceptably high. Many pharmacological and psychosocial interventions for smoking are restricted in accessibility due to barriers such as cost and easy access. This trial has been designed to deliver internet-based response inhibition training in order to offer a simple, low-cost, and easily accessible smoking cessation/reduction intervention. As such, even small effect sizes of the intervention may translate to cumulatively large gains to public health. The current study protocol has been designed to examine the efficacy of response inhibition training to assist dependent smokers to cease or reduce cigarette use.

The intervention has several strengths regarding its timing, delivery and content. Firstly, the intervention maximises the use of being an internet-based program, which capitalises on the ability to have a wide reach within the community at a relatively low cost. This ensures that the intervention is both convenient and highly accessible given that the majority of the population have access to a computer. Secondly, while there is currently limited evidence to suggest that training response inhibition to smoking cues reduces cigarette use or craving [67], previous studies suggest that online response inhibition training to energy-dense food images helps individuals reduce their food intake, weight and food liking [36, 37, 68]. Thirdly, it has been suggested [69] that the best test of stimulus-specific response inhibition training is to use real-world studies that adopt a mixed between- and within-subjects design with repeated-measures (pre to post-intervention). This allows changes from baseline to be computed for meaningful/ecologically valid outcome measures.

While the usual process in translational research is to conduct “proof of concept” studies in the lab before attempting trials in the real-world, we decided to proceed straight to a real-world RCT of smoking-related response inhibition training based on the promising findings in eating behaviour and weight change. This is because laboratory studies can only measure acute training effects that may have little application or predictive value for real-world effects, and because laboratory studies typically adopt a single-session, between-groups design with the dependent variable often being measured only once post-training. This design is limited by confounds such as only one group being exposed to smoking cues during training. Furthermore, if the training relies on changing stimulus-response associations [43], it may be more effective at inducing behavioural change when conducted in real-world contexts associated with smoking (such as the home or workplace) than when conducted in a neutral laboratory setting.

A number of limitations need to be considered. Ongoing studies need to include an objective measure of nicotine use and larger samples in order to adequately power mediation analyses.

### Considerations for future research

If this trial suggests positive effects of smoking-related response inhibition training, future research will need to determine how to optimise outcomes for smokers. Furthermore, research could include examining which aspects of the intervention will produce particular effects. This is both in terms of the training schedule (frequency, duration, timing and location of the training) and the mode of delivery (e.g. online vs. smartphone delivery). Smartphone apps and digital interventions to assist with smoking cessation are very popular but are largely lacking in evidence [70]. This RCT aims to contribute to the evidence-base for the development of new innovative eHealth interventions for smoking cessation.

## Additional file

Additional file 1: **Table S1.** Items from the World Health Organisation Trial Registration Data Set as per SPIRIT guidelines. (DOCX 15 kb)

## Abbreviations

AIH: Automatic Inhibition Hypothesis
ANOVA: Analysis of Variance
AUDIT: Alcohol Use Disorders Identification Test
BIS-11: Barratt Impulsiveness Scale
BSI: Behaviour Stimulus Interaction
CONSORT: Consolidated Standards of Reporting Trials
DASS-21: Depression, Anxiety and Stress Scale
DSM-5: Diagnostic and Statistical Manual of Mental Disorders, Fifth Edition
DUHREC: The Deakin University Human Research Ethics Committee
FTND: Fagerström Test for Nicotine Dependence
GNG: Go/No-go
ID: Identification
INST: Inhibition Smoking Training
NRT: Nicotine Replacement Therapy
RCT: Randomised Controlled Trial
SSD: Stop Signal Delay
SSRT: Stop Signal Reaction Time
SST: Stop Signal Task
T1: Time 1 (baseline assessment)
T2: Time 2 (post-intervention)
T3: Time 3 (one month follow-up)
T4: Time 4 (three months follow-up)
TLFB: Timeline Follow-Back
