# Supplementary material for: A randomised controlled trial examining the efficacy of smoking-related response inhibition training in smokers: a study protocol
Source: BMC Public Health. 2018 Nov 3;18:1226. doi: 10.1186/s12889-018-6109-y (PMC6215605; doi:10.1186/s12889-018-6109-y)
Supplement: Supplementary file 1 — Table S1. Items from the World Health Organisation Trial Registration Data Set as per SPIRIT guidelines. (DOCX 15 kb) [file 12889_2018_6109_MOESM1_ESM.docx]

Additional File 1

Table 1

*Items from the World Health Organisation Trial Registration Data Set as per SPIRIT guidelines*

| **Data Category** | **Information** |
| --- | --- |
| Primary registry and trial identifying number | <http://www.anzctr.org.au/>  [ACTRN12617000252314](https://www.anzctr.org.au/Trial/Registration/TrialReview.aspx?id=370204) |
| Date of registration in primary registry | 16/02/2017 |
| Secondary identifying numbers | Nil |
| Source(s) of monetary or material support | Deakin University, School of Psychology, Faculty of Health.  221 Burwood Highway, Burwood, VIC, 3125, Australia |
| Primary sponsor | A/prof Petra Staiger |
| Secondary sponsor(s) | Dr Melissa Hayden |
| Contact for public queries | Petra Staiger  +61 3 92446876  [petra.staiger@deakin.edu.au](mailto:petra.staiger@deakin.edu.au) |
| Contact for scientific queries | Petra Staiger  +61 3 92446876  [petra.staiger@deakin.edu.au](mailto:petra.staiger@deakin.edu.au) |
| Public title | Evaluating a computer based inhibitory smoking training (INST) program to assist individuals to quit or reduce smoking. |
| Scientific title | A randomised controlled trial examining the efficacy of training response inhibition to smoking in nicotine dependent individuals. |
| Countries of recruitment | Australia |
| Health condition(s) or problem(s) studied | Smoking, addiction, nicotine dependence |
| Intervention(s) | Active comparator: online, Go/No-Go training task using smoking-related images, completed once per day for 14 days.  Control: online, Go/No-Go training task with no smoking images, completed once per day for 14 days. |
| Key inclusion and exclusion criteria | Inclusion criteria: aged between 18-60 years; smoke, on average, a minimum of ten cigarettes per day; meet criteria for moderate or above Tobacco Use Disorder defined by the DSM-5; regular smoker for at least the past 12 months; be motivated to make a quit attempt during the training stage of the intervention; completed at least Year 9 (or equivalent) schooling; have computer and internet access during the intervention phase of the study.  Exclusion criteria: primarily uses electronic cigarettes on a daily basis; non-smoking period of two weeks or more in the past 3 months; currently using anti-craving medication; using nicotine-replacement therapy during the intervention period; self-reported problematic alcohol or drug(s) use other than tobacco; reported a traumatic or acquired brain injury or a loss of consciousness for more than 30 minutes; reported current use of psychotropic medication such as anti-depressant, anti-psychotic and/or anxiolytic medication. |
| Study type | Interventional  Allocation: randomised via computerised sequence generation  Intervention model: parallel.  Masking: double blind  Primary purpose: treatment |
| Date of first enrolment | 22/02/2017 |
| Target sample size | 150 |
| Recruitment status | Stopped early as attrition less than expected |
| Primary outcome(s) | Smoking cessation (time frame: post-intervention; one-month follow-up; three-months follow-up)  Number of cigarettes smoked (time frame: post-intervention; one-month follow-up; three-months follow-up) |
| Key secondary outcomes | Nicotine craving (time frame: post-intervention; one-month follow-up; three-months follow-up)  Self-efficacy (time frame: post-intervention; one-month follow-up; three-months follow-up)  Nicotine dependence (time frame: post-intervention; one-month follow-up; three-months follow-up)  Impulsivity (behavioural and self-report) (time frame: post-intervention; one-month follow-up; three-months follow-up)  Positive valence towards smoking cues (time frame: post-intervention; one-month follow-up; three-months follow-up) |
